# Supplementary material for: Learning curve and surgical outcome of robotic assisted colorectal surgery with ERAS program
Source: Sci Rep. 2022 Nov 29;12:20566. doi: 10.1038/s41598-022-24665-w (PMC9709162; doi:10.1038/s41598-022-24665-w)
Supplement: Supplementary file 1 — Supplementary Information 1. [file 41598_2022_24665_MOESM1_ESM.zip › LSC Raw data-Ñ[▒K/124/H_ñw╝╨░O▒Kñσ.pdf]

正式摘要(電子簽章：000000215255722)<br>W55 / W55 ☐英文標題(注意:無英文姓名-請補病人基本檔!)	☐【讀取】憑證	

臺中榮民總醫院
出院病歷摘要

轉入號 : 轉出號 :

[redacted]

(8)流水編號	
(9)入院日期	110/04/13 (2021) 直腸外科 W55 - 241
(10)轉科日期	
(11)出院日期	110/04/20 (2021) 住院天數計 7 日

診斷

- 入院 :S-colon cancer
- 出院 :S-colon cancer, s/p laparoscopic anterior resection and anastomosis on 2021/4/14 pT3N2b(9/37). stage IIIB

主訴

- bleeding stool

病史

- This is a 51-year-old male with history of:

  1. Anal fistula, s/p fistulectomy
  2. HIVD, s/p OP
  According to the patient, he found bloody stool with diarrhea and was referred from Puli H. S SCOPE 18 CM FAV BX. He was therefore admitted for laparoscopic Anterior resection and anastomosis. He is totally ADL independant. He denies any allergy or family history. He has smoking history.

體檢發現

- S SCOPE 18 CM FAV BX.
  - General appearance :
    - easy looking , conscious clear , without cardiopulmonary distress
    - Vital signs自動帶入date 體溫(BT) 脈搏(P) 呼吸(R) 血壓(BP) 身高(BH) 體重(BW) BMI
      20210413 36.4℃ 66次/分 18次/分 114/82mmHg 167.3cm 92.2kg 32.9
    - Vital signs: B.P. 114/82 mmHg , P.R. 66 /min , R.R. 18 /min , B.T. 6.4 ℃
  - HEENT :
    - Eyes: no anemic conjunctiva, no icteric sclera, normal EOM
    - ENT: no otorrhea, no rhinorrhea, no erythematous mucosa, no injected tonsil, no deformity
  - Neck: supple, freely movable, trachea midline,no deformity, no engorged jugular vein , no palpable thyroid or lymph node enlargement
  - Chest:
    - Inspection: Grossly normal, symmetric and fully expansion, no accessory muscle used, no operation scar, no spider angiomata, no gynecomastia
    - Palpation: no palpable mass or nodule, no subcutaneous emphysema, no tenderness
    - Ausculation: clear breathing sound
  - Heart:
    - Palpation: no thrill, no heave
    - Percussion: normal heart size
    - Ausculation: regular heart beat, normal S1, S2, no gallop, no murmur
  - Extremities: No deformity, full ranged of motion without limitation, no clubbing finger or nail cyanosis, no pitting edema, no ulcer or pigmentation, no obvious skin lesion, no varicose vein, no bone, joint or muscle tenderness

CRS CONDITION

- Karnofsky performance status: (100) Normal, no complaints, no evidence of disease
- PE of Abdomen:
- CEA: 2.33 ng/ml
- Imaging Studies:

-
    - Colonoscope report: 18 FAV
    - Figures:
        1. colon & rectum

手術日期及方法

- 手術日期及方法20210414 Laparoscopic Anterior resection and anastomosis - malignant

住院治療經過

- The patient was admitted for scheduled surgery with laparoscopic Anterior resection and anastomosis. During hospitalization, pre-operative survey showed acceptable conditions. He underwent surgery on 2021/4/14. After surgery, he went back to ward for post-operative care. He presented with sweating and experience a fever episdose after surgery. His fever subsided afterward. Under relatively stable condition, he was therefore discharged with OPD follow-up.

合併症

一般檢查

- 累積報告：

DATE	TIME	ALB	TP	BIL.T	BIL.D	ALKP	AST	ALT	LDH	CHOL	TG	HDL-C	LDL	UA	NH3	AMY
1100413	1556	4.0	6.8	0.4	0.1	84			132							
1100414	1524	3.7					18	25								
1100417	1126			0.84	0.22		15	14								

DATE	TIME	WBC	RBC	HGB	HCT	MCV	PLT	BLST	NEUT	BAND	SEG	LYM	MON	EOS	BAS
1100414	1228	10990	4.75	14.5	42.1	88.6	233		87.9			10.7	0.9	0.1	0.4
1100417	0926	16480		14.0	41.8		215		85.1			8.2	6.3	0.2	0.2
1100420	0857	11530	4.33	13.1	39.6	91.5	293		80.7			11.5	6.2	1.4	0.2

DATE	TIME	NA	K	CL	CA	BUN	CREAT	Mg	P	CRP	ESR	PCT	LIPASE	LACTATE
1100414	1524	140	4.3			6	0.98							
1100417	0903													7.3
1100417	0930											0.074		
1100417	1126	138	3.7			6	0.97							

DATE	TIME	SP-GR	PH	PROT	GLUC	KETON	BILI	UROB	OB	NITR	LEU	RBC	WBC	EPCEL
1100418	0055	1.020	7.5	Trace	Negative	1+	Negative	0.2	Negative	Negative	Negative	0-2	0-2	0-1

特殊檢查

病理報告

其它

出院時情況

- 3.改門診治療

出院指示

-

藥名	劑量	劑量單位	用法	頻次	數量	數量單位	現況	天數	單位
(□)Takepron OD tab 30mg	1	TAB	PO	QDAC	7	TAB	TH	7	天
Ultracet tab	1	TAB	PO	Q6HPRN	12	TAB	TH	3	天
Mag. Oxide tab	2	TAB	PO	TID	42	TAB	TH	7	天
U-Clor cap 250mg	2	CAP	PO	Q8H	30	CAP	TH	5	天

出院計劃

主治醫師_2222H 林俊余 / 住院醫師_9958C 林強生

正式摘要(電子簽章：000000215255722)

W55 / W55

doctorRank:B
